# Fabrication and performance evaluation of polyethersulfone membranes with varying compositions of polyvinylpyrrolidone and polyethylene glycol for textile wastewater treatment using MBR

**DOI:** 10.1016/j.heliyon.2024.e36215

**Published:** 2024-08-16

**Authors:** Md. Nur-E Alam, Shamim Ahmed Deowan, Shakil Shahriar Efty, Fariha Chowdhury, Ahsanul Haque Milon, Mohammad Nurnabi

**Affiliations:** a Department of Applied Chemistry and Chemical Engineering, University of Dhaka, Dhaka, 1000, Bangladesh; b Leather Research Institute (LRI), Bangladesh Council of Scientific and Industrial Research (BCSIR), Savar, Dhaka, 1350, Bangladesh; c Department of Robotics and Mechatronics Engineering, University of Dhaka, Dhaka, 1000, Bangladesh; d Biomedical and Toxicological Research Institute, Bangladesh Council of Scientific and Industrial Research, (BCSIR), Dhaka, 1205, Bangladesh; e Institute of Leather Engineering and Technology, University of Dhaka, Dhaka, 1209, Bangladesh

**Keywords:** Textile wastewater, Phase inversion, PEG, PVP, PES

## Abstract

Various industries polluting the water bodies by discharging untreated wastewater directly into the environment and conventional wastewater treatments are often insufficient for effectively treating the pollutants. However, membrane bioreactors (MBRs) offer a promising solution for wastewater treatment where membrane serving as the heart of the system. In this study, polyethersulfone (PES) was used as the membrane material and hydrophilicity of the membranes were tuned up by mixing with hydrophilic additives such as polyethylene glycol (PEG) and polyvinylpyrrolidone (PVP) and the membranes have shown promising results in treating wastewater, particularly in terms of chemical oxygen demand (COD), biochemical oxygen demand (BOD), and color removal. For example, PES-PEG membrane demonstrated COD, BOD, and color removal of 96 %, 94 %, and 92 %, respectively while those were 95 %, 94 %, and 92 %, respectively for PES-based commercial membrane. Although the performances of fabricated membranes were comparable to that of commercial membrane in COD, BOD, and color removal efficiencies, there is room for improvement in permeate yields. Notably, the average permeate efficiency for MBR modules produced with PES-3PEG and PES-5PVP membranes was recorded as 47 % (18 L/m^2^h) and 13 % (5 L/m^2^h) respectively of the commercial membrane (38 L/m^2^h). Despite the variance in permeate yields, the fabricated membranes also showcased significant efficacy in removing microorganisms, a crucial aspect of wastewater treatment. Their performance in this regard proved highly comparable to that of the commercial membrane, emphasizing the potential of these fabricated membranes in enhancing the wastewater treatment.

## 1 Introduction

Membrane technology plays a crucial role in advancing efficient and sustainable wastewater treatment solutions. Acting as effective barriers, membranes selectively eliminate contaminants from wastewater through techniques such as microfiltration, ultrafiltration, nanofiltration, reverse osmosis, etc [1,2]. However, a significant challenge arises as most of the membrane materials are relatively hydrophobic, attract and retain hydrophobic contaminants, leading to membrane fouling [3]. However, membrane fouling can also be caused by biological, colloidal, and organic materials. To address this issue, the membrane fabrication process often incorporates various hydrophilic additives through blending, coating, surface grafting, etc [4]. Blending of PES polymer solution with hydrophilic polymers effectively enhances membrane hydrophilicity and minimizes fouling risk [5]. In this study, hydrophilic additives, namely PEG and PVP, were integrated in the fabrication of polyethersulfone (PES) membranes using non-solvent induced phase separation (NIPS) process. In NIPS process, a uniform polymer dope solution with or without additives transforms into a two-phase system, generating a solid polymer-rich phase as the membrane matrix and a liquid polymer-poor phase to form the membrane pores [6]. Polyethersulfone (PES) porous membranes are highly examined for liquid and gas separation due to their selective permeability. Optimizing blend composition and coagulation conditions is essential for developing membranes with specific properties. Blending PES with hydrophilic polymers enhances hydrophilicity and reduces fouling of the membrane. The role of hydrophilic polymers goes beyond tailoring porosity, they also provide hemocompatibility, cytocompatibility and antifouling properties, thereby enhancing the use of these membranes in biological separation [7]. As pore forming agent, PEG is widely used in the membrane fabrication and its incorporation not only enhances the flux and hydrophilicity of fabricated membranes but also imparts anti-fouling properties [8,9]. It also acts as a barrier of macrovoids formation, increasing membrane hydrophilicity, and permeability [10]. PVP is also a pore forming agent, which is used in both flat sheet and hollow fiber membrane fabrication and it is compatible with many membrane materials [11]. It increases the permeability by enhancing membrane porosity without significantly altering selectivity [12]. The characterization of fabricated membranes involved the use of Fourier-transform infrared (FTIR), scanning electron microscope (SEM), contact angle measurement, pure water flux, and porosity assessments. Notably, PEG blended membranes exhibited higher pure water fluxes, along with a higher percentage of porosity compared to PVP blended membranes.

The global population surge has driven industrial expansion, increasing the demand for abundant water supplies and high-quality effluent through advanced treatment technologies. Membrane bioreactors (MBRs) address these needs by effectively removing organic and inorganic matter in wastewater treatment [13]. These processes combine biological and filtration processes for effective wastewater treatment which are superior in performance over suspended growth system, providing advantages in solids and organic removal, a smaller footprint, high loading rates, and minimal sludge production. However, fouling hinders the filtration efficiency, leading to increased operational costs [14]. Over the last two decades, global studies on MBRs have risen sharply to meet strict discharge standards and address the increasing demand for wastewater reuse by enhancing effluent quality, leading to extensive academic attention and rapid growth in practical applications worldwide [15,16]. MBR replaces the sedimentation tank of the conventional activated sludge process, operating with a higher SRT, which results in the effective retention of most bacteria within the reactor that contributes to improved treatment efficiency [17]. Deng et al. investigated the use of a bioflocculant in an MBR for municipal wastewater treatment and found that it reduced soluble microbial products in the suspended sludge, thereby alleviating membrane fouling [18]. Corpuz et al. studied various MBR processes, including the Algae-Activated Sludge Membrane Bioreactor (AAS-MBR) and electro Algae-Activated Sludge Membrane Bioreactor (e-AASMBR). They observed similar chemical oxygen demand (COD) reduction in both processes. However, nutrient removal, including phosphate phosphorous, and ammoniacal nitrogen, was higher in the AAS-MBR compared to the e-AASMBR [19]. Combination MBR with other membrane processes are also gaining popularity. Lie et al., integrated NF with MBR for textile wastewater treatment and achieved high water recovery through the recirculation of nanofiltration concentrate to the MBR in the MBR-NF hybrid process [20]. Lin et al., investigated hybrid MBR-RO system for reuse of textile wastewater in manufacturing processes demonstrating the high feasibility of combining MBR with RO for its treatment and reclamation [21].

In recent decades, Bangladesh has seen a substantial increase in textile, tanning, and manufacturing industries, generating large amounts of wastewater. Among them, the textile industry produces large volumes of highly polluted and toxic wastewater. Over 100,000 textile dyes are commercially available with annual production ranging from 700,000 to 1,000,000 tons. Of this, around 280,000 tons are discharged into the environment through effluents from the textile industry. According to the World Bank, the textile industry accounts for 17–20 % of industrial water pollution globally [22]. However, despite the environmental regulations, these sectors frequently discharge wastewater without proper treatment causing adverse environmental effects. The use of MBRs in the textile sector is rare and research on this technology in Bangladesh is limited.

Finally, the fabricated membranes with the best results were employed in a membrane bioreactor (MBR) and their performances were compared with that of a commercial membrane for treating synthetic textile wastewater. A bacteriological analysis was also performed before and after the treatment. To the best of our knowledge, there is limited research dedicated to the concurrent development of membranes and their integration into membrane bioreactor (MBR) for the treatment of textile wastewater. The main objectives of this study was to fabricate low-cost PES membranes blended with PEG and PVP additives, and also compared the results with the PES-based commercial membrane.

## 2 Experimental

### 2.1 Materials for membrane fabrication

**Fig. 1:**
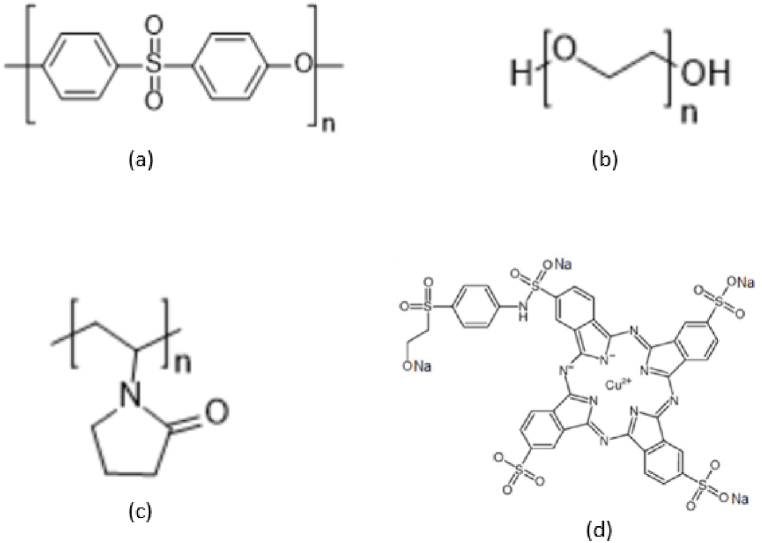
Chemical structures of PES [23] (a), PEG [24] (b), PVP [23] (c), and (d) C.I. Reactive Blue 21 dye [25].

Polyethersulfone (PES-58K, nominal granule size 3 mm), polyvinyl pyrrolidone (PVP–40K), and polyethylene glycol (PEG-6k) were purchased from Goodfellow Cambridge Ltd. UK, Sigma-Aldrich, and Research-Lab, India, respectively. Dimethylformamide (DMF) M.W. 73.09 g/mol was bought from Daejung Chemicals and Metals Co. Ltd, Korea. Non-woven Holytex-3256 was bought from TALAS, USA. PES-based commercial membrane was purchased from Microdyn-Nadir company, Germany, with MWCO 150 KDa. Turquoise blue (C.I. Reactive Blue 21) with molecular weight of 1167.5 g/mol was supplied by a local dye supplier. All chemicals and materials were used as received. Fig. 1 shows the chemical structures of PES (a), PEG (b), PVP (c), and (d) C.I. Reactive Blue 21 dye.

### 2.2 Fabrication of membrane by non-solvent induced phase separation (NIPS) process

#### 2.2.1 Formulation of dope solution

To formulate a 100g dope solution, a specific quantity of PES (15–19 wt%), PEG (1–7 wt%), PVP (1–7 wt%) and DMF (80-74 wt%) were taken in a closed glass container. The mixer was thoroughly stirred on a hotplate magnetic stirrer at 80°C for 4–5 h [26]. Subsequently, the solution was left to stand overnight at room temperature followed by 1-h sonication process to eliminate any trapped air bubbles. The composition of dope solutions is detailed in Table 1.

**Table 1:** Composition of dope solution.

Code	PES (wt.%)	PEG (wt.%)	PVP (wt.%)	Solvent (DMF wt. %)
15 PES	15	–	–	85
17 PES	17	–	–	83
Control	19	–	–	81
1 PEG	19	1	–	80
3 PEG	19	3	–	78
5 PEG	19	5	–	76
7 PEG	19	7	–	74
1 PVP	19	–	1	80
3 PVP	19	–	3	78
5 PVP	19	–	5	76
7 PVP	19	–	7	74

#### 2.2.2 Casting of the dope solution

**Fig. 2:**
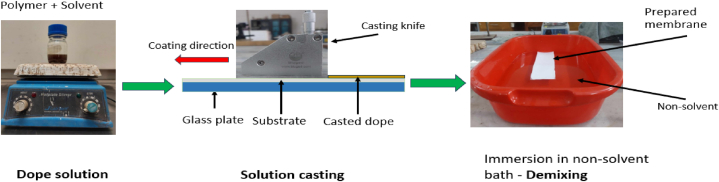
Schematic view of membrane fabrication by NIPS process.

The dope solutions were applied onto a membrane support material fixed on glass plate using a casting knife, maintaining a clearance of 250 μm. The cast solutions were allowed to leave for 3–5 s in ambient atmosphere (26–30^**o**^C and relative humidity of 50–65 %). Subsequently, they were dipped in a non-solvent bath containing distilled water for demixing. The resulting membranes were then submerged in distilled water for 24 h to thoroughly eliminate any remaining solvent. The membrane fabrication process is illustrated in Fig. 2.

### 2.3 Characterization of fabricated membranes

#### 2.3.1 Effects of polymer concentration on membrane skin formation

Blended membranes were fabricated by incorporating different proportions of PES with varying amounts of additives such as PEG and PVP. Non-woven Hollytex-3256 fabric was used as the supporting material for these membranes. The examination of surface of the fabricated membranes was conducted using tinyscope 1000x mobile microscope.

#### 2.3.2 Analytical tools

Fourier-transform infrared (FTIR) spectra, conducted with attenuated total reflectance (ATR) were recorded using the IR Prestige-21 model, Shimadzu, Japan to identify the various functional groups within the membranes. The morphology of the membranes was investigated through the use of a field-emission scanning electron microscope (FE-SEM) (Model: JSM-7610F, Japan). This technique provided direct insights into the surface morphology of the membranes. For FE-SEM, sampling preparation, the samples were sputtered with platinum (1 nm thickness). For pore size measurement using SEM images, ImageJ software was use.

#### 2.3.3 Equilibrium water content (EWC)

The determination of equilibrium water content involved 2 cm × 2 cm cut pieces of membrane and weighted in an electronic balance. Subsequently, these cuts were then dipped in distilled water for a duration of 24 h. After that the membrane pieces were mopped carefully with tissue paper to remove excess water from the surfaces and weighted again. The percentage of water uptake was then calculated using Eq. (1) [27].

(1) $$\text{EWC}\left(\%\right)=\frac{W0-W1}{w1}\times 100$$

where, W_0_ and W_1_ (g) are the masses of the wet membrane and the dry membrane, respectively.

The overall porosity of the membranes (*ε*) was determined by the gravimetric method according to following Eq. (2) [28].

(2) $$\varepsilon \left(\%\right)=\frac{W0-W1}{A\times l\times dw}\times 100$$

Where, d_w_ is the density of water (g/cm^3^), A is the membrane effective area (cm^2^), and l is the thickness of membranes (cm).

#### 2.3.4 Contact angle measurement

**Fig. 3:**
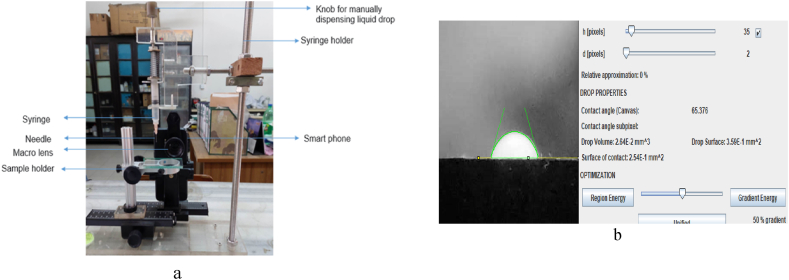
In-house developed contact angle device (a) and image analysis conducted using ImageJ software (b).

In this study, an economical device for measuring contact angles was developed as depicted in Fig. 3a. It utilized a Samsung A52s smartphone, a macro lens (Apexel APL-HB 100 mm), a mobile phone holder, a sample holder, and a 3.5 cc plastic syringe for dispensing water droplets. The sessile drop was controlled using a knob located on the syringe's top. To determine the membrane contact angle, a water droplet was applied to the membrane surface, and the contact angle was recorded between 10 and 30 s [29,30]. To ensure accuracy, multiple images were captured at different points, and average values were calculated. The contact angle of water droplet on the membrane surface was analyzed using ImageJ Software (Fig. 3b).

#### 2.3.5 Pure water flux (PWF)

Crossflow filtration unit (OSMO, Germany) was employed to assess the pure water flux of the fabricated membranes, with a membrane filtration active area of 0.008 m^2^ and operating pressure set at 3 bar to drive the distilled water through the membrane. The permeate flux (expressed in L/m^2^h) was determined by the following Eq. (3) [27].

(3) $$\text{Jp}=\frac{Vp}{A\times \Delta t\;}$$

where *Jp* = permeate flux (LMH), *Vp* = volume of permeate (L), *A* = area of the membrane (m^2^) and Δt = time (h) required to collect permeates at different transmembrane pressures.

#### 2.3.6 Experimental set-up and operation of MBR

**Fig. 4:**
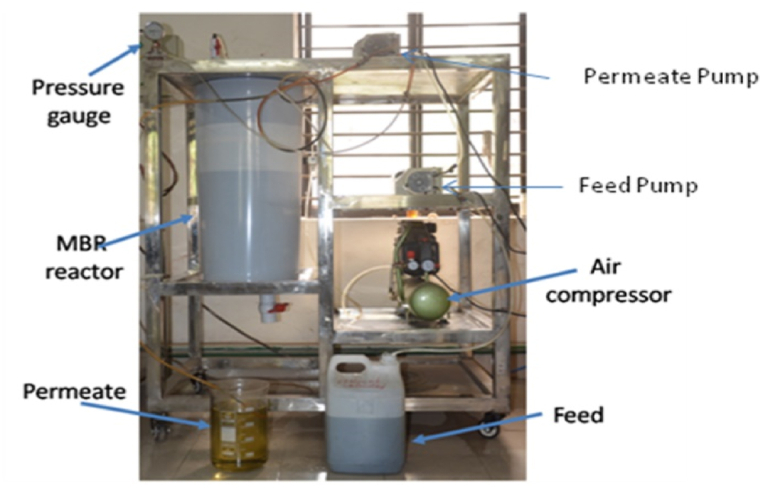
Image of submerged membrane bioreactor (SMBR).

Sludge was collected from Flagship Dhaka Common Effluent Treatment Plant (CETP), Dhaka Export Processing Zone (DEPZ), Savar, Bangladesh. As the characteristics of real textile effluent change dramatically from day to day, a synthetic textile effluent with composition similar to real effluent was prepared based on the information presented in Table 2. A laboratory scale (50 L) submerged membrane bioreactor (SMBR) was used to run the whole experiment as shown in Fig. 4. Two flat sheet fabricated membranes with effective surface area of 0.025 m^2^ were directly immersed in the tank. The sludge was acclimated using synthetic textile wastewater to get MLSS concentration of 4–6 g/L. Around 2–3 mg/L dissolved oxygen (DO) was maintained throughout the experiment. Aeration (560 L air/m^2^ membrane area) serves a threefold purpose: aeration, mixing of the biomass, and cleaning of the membrane [31]. The characteristics of the final MBR feed were presented in Table 3.

**Table 2:** Composition of synthetic textile wastewater [33].

Chemicals	Concentration (mg/L)
Turquoise blue dye (C.I. Reactive Blue 21)	100
Ammonium Chloride (N-source)	300
Sodium Chloride (as electrolyte)	2500
Sodium bicarbonate (as pH buffer)	1000
Detergent	50
Glucose (C-source)	2000

**Table 3:** Characteristics of MBR feed.

Chemicals	Concentration (mg/L)
COD (mg/L)	6500
BOD (mg/L)	3100
pH	7.66
Turquoise blue dye (C.I. Reactive Blue 21)	94
TDS	3600
MLSS	4000–5000

Chemical oxygen demand (COD) and Biochemical oxygen demand (BOD) were analyzed based on the Standard Methods [32]. Color was measured by spectrophotometric method (UV-1700 PharmaSpec, Shimadzu) by measuring absorbance at 592 nm which is the *λ*max of the used dye. The dye concentration was estimated by calibration method where a calibration curve was constructed in advance.

In Table 3, the dye concentration of the MBR feed decreased due to the addition of the sludge to the synthetic wastewater.

#### 2.3.7 Bacteriological analysis

Samples from before and after the operation of each membrane were collected from the MBR plant and poured into a sterile tube. All the samples were brought to the laboratory by maintaining temperature (4–8 °C) using a cool box within 2 h of collection. Microbiological analyses were done according to USFDA Bacteriological Analytical Methods (2001). The bacteriological analysis was carried out by serially diluting the samples and plating 1.0 mL of the appropriate dilution onto Tryptic Soy Agar (Fluka, USA) microbial culture media for total aerobic bacterial count; Hichrome^TM^ Coliform Agar (Fluka, USA) media for total coliform bacterial count; Sorbitol MacConkey Agar (Oxoid, UK) medium for enumerating fecal coliform bacterial count; NGKG agar: Kim and Goepfert agar with NaCl and Glycine for *Bacillus* spp. and Cetrimide selective agar for *Pseudomonas* sp 37 °C for 24 h and Dichloran Rose Bengal Chloramphenicol Agar for total yeast and mold count. After inoculating the samples, all culture media plates were incubated for 24–48 h at 37 °C before being counted. All the plate count data were represented as the mean values obtained from three individual trials, with each of these values being obtained from duplicated samples.

## 3 Results and discussion

### 3.1 Effects of PES concentration on membrane skin formation, FTIR analysis and SEM

**Fig. 5:**
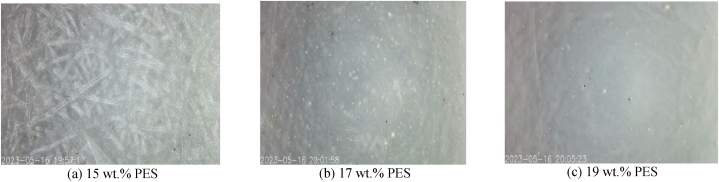
Effect of polymer concentration on membrane skin formation (magnification was done tinyscope 1000x mobile microscope).

Polymer solutions with concentration ranging from 15 to 19 wt% were cast onto the support material. It was observed that only the 19 wt% polymer concentration yielded a consistently uniform skin on the support material as shown in Fig. 5c. Conversely, a membrane with a 15 wt% concentration of polymer revealed the fiber of the support material. This depicted in the magnified image of Fig. 5a. It could be inferred from Fig. 5c that at 19 wt% the viscosity of the cast solution was enough to form a uniform film on the support material while below this concentration, the cast solution did not cover the support material efficiently.

**Fig. 6:**
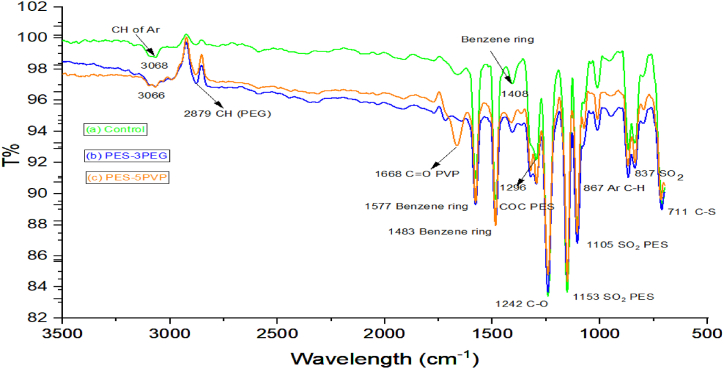
FTIR/ATR spectra of (a) Control membrane, (b) blended PES-3PEG membrane (e) blended PES-5PVP membrane.

FTIR/ATR spectra of PES-control membrane, PES-3PEG, and PES-5PVP blended membranes are illustrated in Fig. 6. In PES-control membrane distinct peaks at 1577 cm^−1^, 1483 cm^−1^, and 1408 cm^−1^ corresponds to the presence of aromatic ring. Peak 3068 cm^−1^ and 1242 cm^−1^ correspond to the presence of aromatic C–H and C–O group while the peak at 1296 cm^−1^ represented ether group (C–O–C). The sulfonyl group is identified through two stretching vibration at 1153 cm^−1^, 1105 cm^−1^, and 837 cm^−1^. These spectral features of PES is aligned well with the previous studies on PES FTIR [[34], [35], [36]].

Similar to PES-control membrane, the presence of aromatic rings in PES-3PEG (Fig. 6b) and PES-5PVP (Fig. 6c) membranes were identified by peaks around 3030-3100 cm^−1^, corresponding to aromatic C–H stretching vibrations and characteristics stretching vibrations of aliphatic C–H bond were observed in all the membranes at around 2800 to 3000 cm^−1^. For PES-5PVP membrane a strong C=O stretching group was observed at 1668 cm ^−1^ (Fig. 6c).

**Fig. 7:**
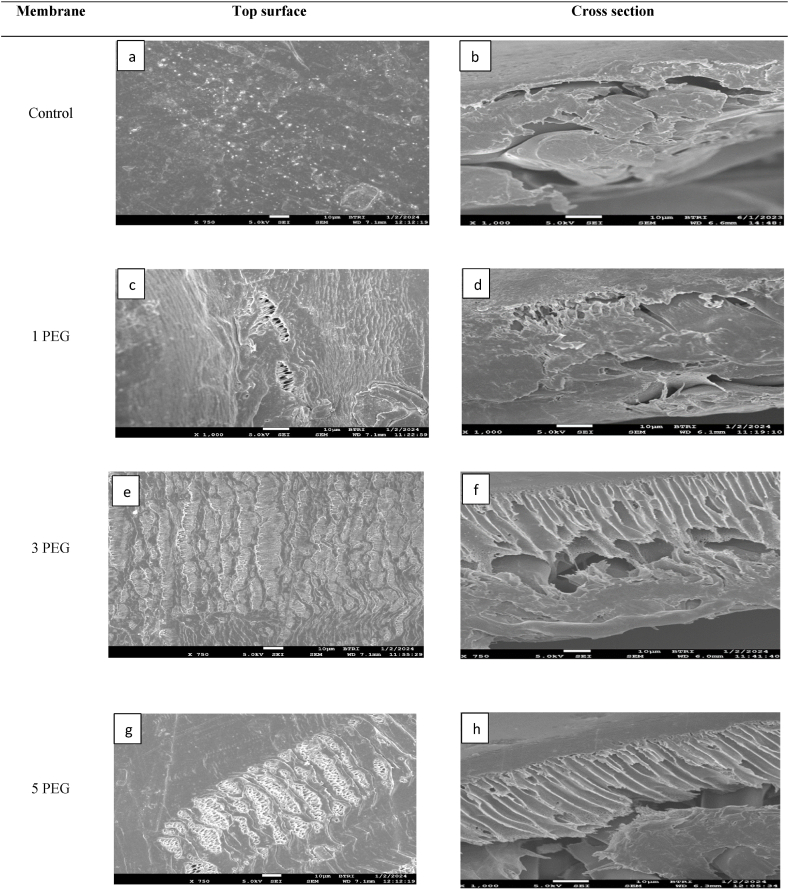

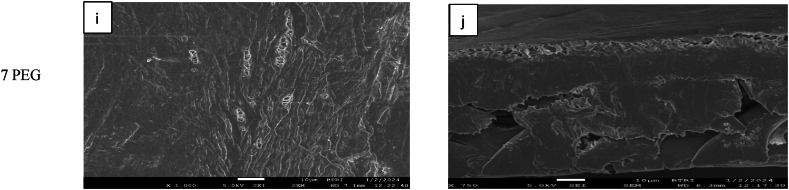
FE-SEM images of Control and PEG blended membranes, (Left) surface view and (Right) cross section view.,

Fig. 7 illustrates the FE-SEM images of PES-control and PES-PEG blended membranes. The PES-control membrane exhibited a top dense layer (Fig. 7b) with nonporous surface (Fig. 7a). The introduction of PEG additive enhances the number of pore and pore sizes up to 3 wt%. Beyond this point, pore size decreased, which is attributed to the escalating viscosity of the dope solution. It was inferred that up to a 3 wt% concentration of PEG, the diffusion rate of non-solvent and solvent exchange is optimized, leading to instantaneous demixing and the eventual formation of a porous membrane. However, at higher additive concentration, the increased viscosity of the dope solution hinders the process, causing delayed demixing and a subsequent reduction in membrane porosity [10].

**Fig. 8:**
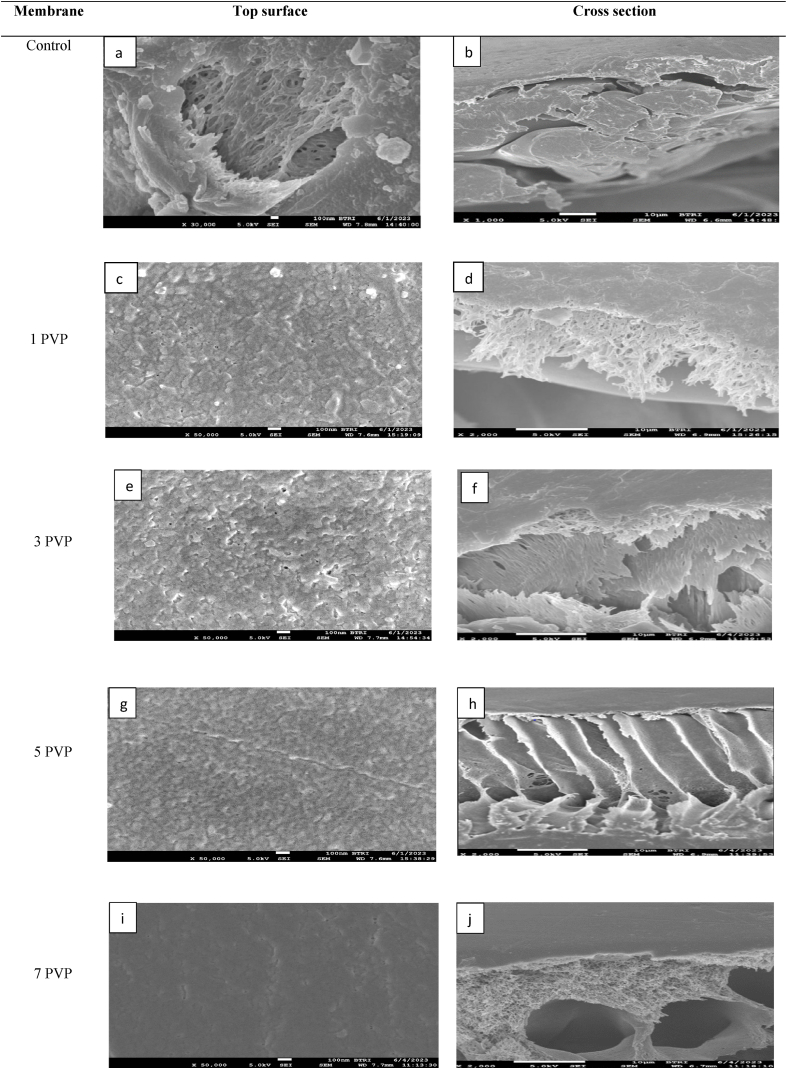
FE-SEM images of PVP blended membranes, (Left) surface view and (Right) cross section view.

Fig. 8 shows the FE-SEM images of PES-PVP blended membranes. In the case of PES-PVP blended membranes, both surface and the cross-section images reveal dense membrane structures. Due to its higher molecular weight relative to PEG molecule, PVP is less readily washed out during the demixing process requiring an extended period for the additive to be effectively removed from the membrane film. This prolong duration allows polymer aggregates to accumulate on the top layer making a non-porous membrane [37].

### 3.2 Pore size measurement, equilibrium water content, membrane porosity, pure water flux, and contact angle

**Fig. 9:**
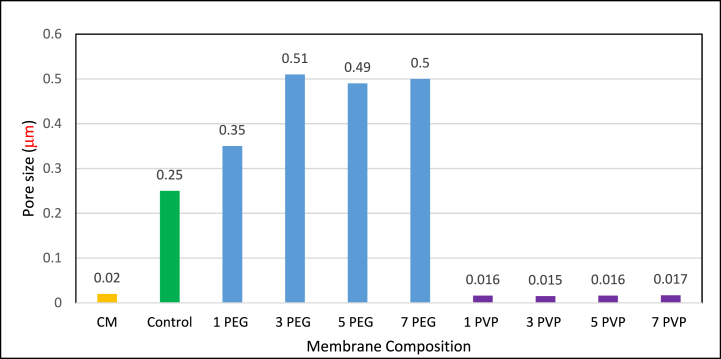
Pore size distribution of fabricated membranes.

Fig. 9 shows the pore sizes (μm) of commercial (CM) and different fabricated membranes. CM and control membranes had average pore size of 0.02 μm and 0.2 μm, respectively while PES-PEG membranes showed increased pore size reaching its maximum of 0.51 μm at 3 wt% PEG concentration. However, a further increase in PEG concentration had a minimal impact, as it led to an elevation in dope solution viscosity that consequently producing a membrane with suppressed pores [10]. For PES-PVP membranes, the pore sizes were in the range of 0.015–0.017 μm closed to the commercial membrane. At respective optimum concentrations, the average pore size of PES-PEG blended membrane was approximately 3000 times larger than that of PES-PVP blended membrane. It was assumed that PEG functioned as a more efficient pore-forming agent compared to PVP which may be achieved through PEG's role as a plasticizer that facilitated the expansion of the polymer matrix and the subsequent formation of pores [38].

**Fig. 10:**
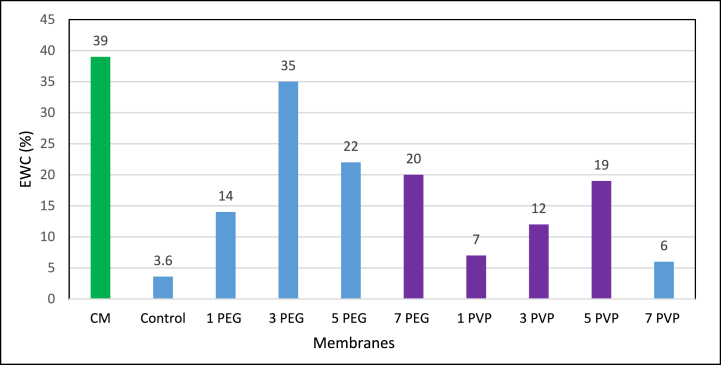
Equilibrium water content (%) of fabricated membranes.

Equilibrium water content (EWC) is a key membrane characterization parameter, assessing the membrane's hydrophilicity which in turn influences water flux and porosity. This represents the fraction of voids space relative to the apparent total bulk volume of the material [39]. EWC of various fabricated membranes are presented in Fig. 10. With the incorporation of PEG and PVP additives, the EWC increased compared to the PES-control membrane. This rise in water content confirms the presence of a greater number of pores in the fabricated blended membranes than that of the PES-control membranes due to incorporation of these additives. Fabricated PES-3PEG showed about 84 % higher EWC than the PES-5PVP membrane. However, all the fabricated membranes showed lower EWC than the commercial membrane (CM). On average, the fabricated PES-3PEG and PES-5PVP membranes exhibited 10 % and 70 % less EWC, respectively, compared to the CM membrane.

**Fig. 11:**
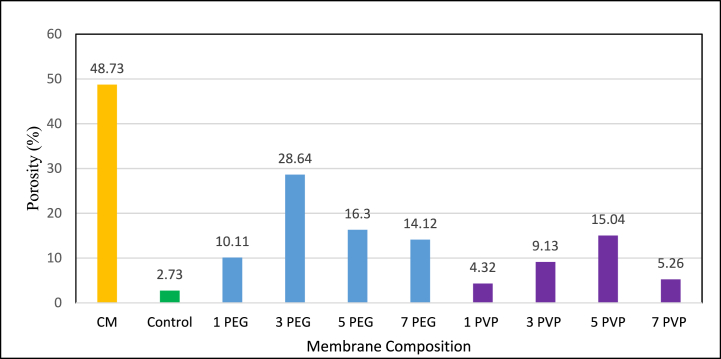
Percentage of porosity of fabricated membranes.

The porosity of fabricated membranes is depicted in Fig. 11. For PEG and PVP blended membranes, porosity steadily increased up to 3 % PEG and 5 % PVP additive concentration. This enhancement was attributed to the hydrophilic nature of PEG and PVP, facilitating solvent/non-solvent mass transfer and resulting in highly porous structure. However, beyond these additive concentrations, porosity declined due to increased viscosity which hindered the coagulation process, leading to reduced porosity in the membrane [40]. Fabricated PES-3PEG exhibited about 91 % higher porosity than the fabricated PES-5PVP membrane.

Water-soluble additives (e.g., PEG, PVP etc.) have been proven to serve as pore formers in phase separation membrane fabrication processes. The presumption is that these water-soluble additives can be leached out from the casting film, generating micropores in the location they once occupied [41,42]. The hydrodynamic radius of PEG and PVP are 1.8 nm and 5.1 nm, respectively [43,44]. For that reason, PEG molecule can easily leach out from the casting film during the demixing process and producing more porous structures than PVP blended membranes. However, commercial membrane (CM) exhibited 70 % and 224 % higher porosity compared to the fabricated PES-3PEG and PES-5PVP membranes, respectively. The higher percentage of porosity of PEG blended membranes can also be explained with the help of solubility parameter shown in table (S1). From the Table S1, it can be inferred that PEG can easily dissolve in water more readily than PVP due to its closeness of solubility parameter to that of water. Hence, during the demixing process, PEG can easily be washed out or leached into the non-solvent water bath leaving behind pores in the membrane. This phenomenon enhances instantaneous phase separation ultimately resulting in the formation of a porous membrane [45, 46].

**Fig. 12:**
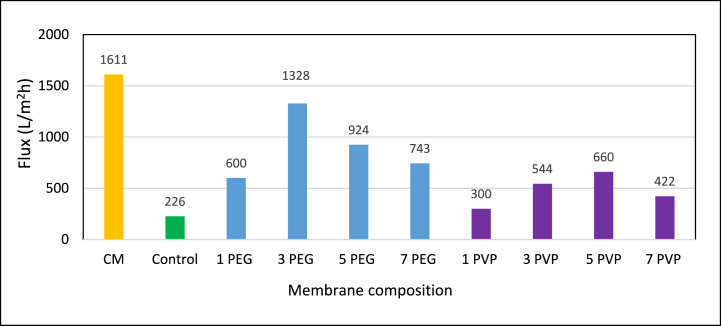
Effects of additive concentrations on pure water flux at 3 bar.

The pure water flux (L/m^2^h) of fabricated membranes was determined through crossflow filtration unit conducted at a pressure of 3 bar as depicted in Fig. 12. The results indicated a discernible impact of additive concentration in the casting solution on the water flux. The pure water fluxes increased up to 3 % of PEG additive concentration and 5 % of PVP additive concentration. Membranes fabricated with these additive concentrations exhibited higher porosity leading to increased flux and beyond these additive concentrations, the fluxes of both types of blended membranes declined. This could be ascribed to the increased viscosity of the casting solution, leading to a reduction in the rate of solvent and non-solvent interchange during the demixing process, thereby causing delayed demixing.

This phenomenon led to the formation of a dense top layer (SEM Fig. 7, Fig. 8) with suppressed pore sizes [10]. It was also observed that the pure water flux of PES-3PEG membranes was 101 % higher flux than the PES-5PVP. This disparity was attributed to the higher porosity exhibited by the PES-3PEG blended membrane. However, CM membrane showed approximately 21 % and 144 % higher fluxes compared to the PES-3PEG and PES-5PVP membrane, respectively.

**Fig. 13:**
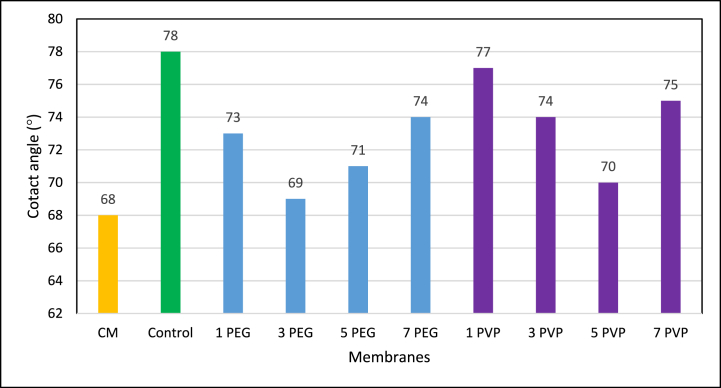
Contact angle of fabricated membranes (average contact angle of five replicates are reported).

The contact angle, a visual measure of a liquid droplet on a surface, aids in assessing the surface tension between the liquid and solid substrate. A high contact angle indicates elevated surface tension between the solid and liquid, while a low angle suggests the opposite. When the contact angle is less than 90°, the membrane demonstrates hydrophilic characteristics, whereas a contact angle exceeding 90° leads to hydrophobic behavior of the membrane [47]. Contact angles of PES-control and blended membranes were assessed using the in-house built contact angle device shown in Fig. 13.

The figure illustrates that the incorporation of PEG and PVP additives to the dope solution resulted in decreased contact angles of the fabricated membranes compared to the control membrane. Both the PEG and PVP additives are hydrophilic in nature and contributed to the reduction in contact angles for the blended membranes, showing similar trends. However, when the addition of PEG exceeded 3 wt% and PVP exceeded 5 wt%, the contact angles increased again. This rise in contact angle values connected with the decrease in porosity attained at higher dope solution viscosity [48].

Based on these characteristics, fabricated PES-3PEG and PES-5PVP membranes were selected for use in the MBR for textile wastewater treatment and their results were compared with the commercial PES-based membrane (CM).

### 3.3 MBR performance

**Fig. 14:**
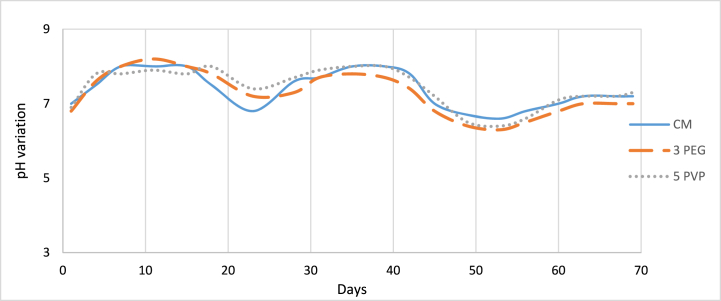
Change of permeate pH.

Membrane bioreactor equipped with one commercial membrane (CM) and two fabricated membranes, PES-3PEG and PES-5PVP (selected on the basis of pure water fluxes). The treatment of synthetic textile wastewater was running for around 70 days. Fig. 14 illustrates the variation of pH of the permeates with times and it was evident that both the CM and fabricated membranes showed similar trends. In biological wastewater treatment, aerobic bacteria consume organic pollutants in the presence of oxygen converting them into carbon dioxide, water, and new cells. This carbon dioxide can dissolve in water in the MBR tank leading to the formation of carbonic acid and subsequently causing a decrease in pH. Since MBR system cannot take advantage of SRT longer than 30 days [49], the lowest pH levels are typically observed between the 20 and 30 days. This timeframe is assumed to coincide with optimal biological activity, resulting in enhanced biodegradation and increased carbon dioxide production. Incorporation of new sludge after 30 days of operation, pH was again dropped which was between 45 and 50 days.

#### 3.3.1 BOD and COD removal

**Fig. 15:**
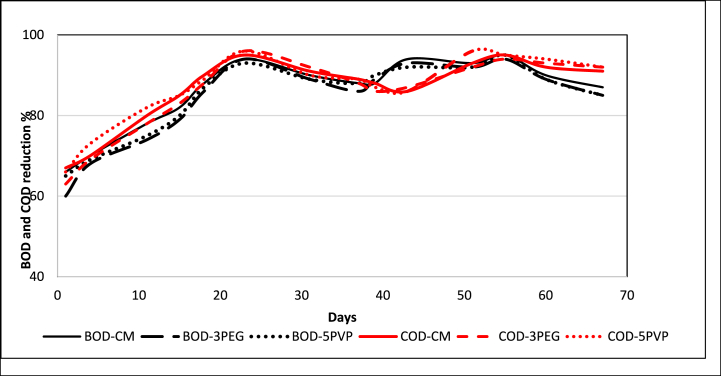
Percentage of BOD (black color) and COD (red color) removal. (For interpretation of the references to color in this figure legend, the reader is referred to the Web version of this article.)

The results of BOD and COD removal for the synthetic textile wastewater are presented in Fig. 15. It was found that the removal efficiency for the organic matter by MBR was very high for all the applied membranes. About 93–94 % of BOD and 95–96 % of COD were removed after 25 days of operation, then dropped a bit, due to loss of activity of microorganisms. The addition of new sludge (after 30 days) again increased the BOD and COD removal. MBR does not benefit from prolonged SRT exceeding 30 days in terms of active biomass. Instead, the heightened concentration of solids may exacerbate the fouling tendency of the membranes [49]. Therefore, following 30 days of continuous operation, fresh active sludge was introduced into the MBR tank that provided active microorganisms for the organic matters and a renewed increase of BOD and COD removal was achieved. It was noteworthy that fabricated membranes demonstrated equivalent efficiencies to commercial membrane in removing organic matter from wastewater using MBR. Moreover, in MBR operation, mixed liquor suspended solid (MLSS) and solid retention time (SRT) play vital role in degradation of organic matters and thus high MLSS and high SRT are generally maintained.

In this study, MLSS of 4–5 g/L and SRT of 30 days were maintained. Apart from biodegradation, it was reported that filtration alone contributes approximately 30 % to the overall BOD and COD removal process [50]. In wastewater, organic matter can be divided into dissolved organic carbon (DOC) and particulate organic carbon (POC). DOC typically ranges in size from 10^−2^ to 10^−4^ μm, while POC ranges from 0.1 to 100 μm [51]. Although MBR is not efficient enough to remove dissolved organic matter, it can effectively separate particulate matter from the effluent due to its pore size range.

#### 3.3.2 Color removal (%)

**Fig. 16:**
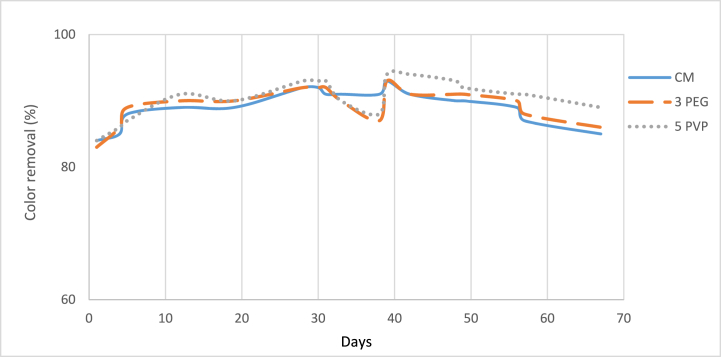
Percentage of color removal. (For interpretation of the references to color in this figure legend, the reader is referred to the Web version of this article.)

Fig. 16 illustrates the variation of dye removal (%) with operation days and the initial dye concentration was 94 mg/L. It was evident that the PES-5PVP blended membrane demonstrated superior performance, achieving the highest color removal of 94 % while CM and PES-3PEG blended membranes exhibited 93 % color removal after 39 days of operation. The percentage of color removal showed a steady increase over the course of 30 days, followed by a declining trend up to day 38. Subsequently, there was another increased observed. This fluctuation can be attributed to the introduction of new activated sludge after 30 days of operation. It may take some days for this new sludge to adapt to the wastewater in the MBR system, leading to an eventual improvement in color efficiency.

**Fig. 17:**
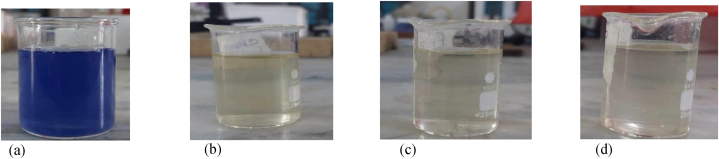
Images of (a) Feed water (b) permeate from CM membrane (c) PEG blended membrane, and (d) PVP blended membrane.

MBR uses microfiltration or ultrafiltration membranes, which primarily serve to physically separate solids from liquids in wastewater. These membranes do not directly remove color from the wastewater. The primary mechanism for color removal is adsorption onto biomass. However, the biodegradation of textile dyes is limited in activated sludge systems due to their persistent nature [22,52]. Additionally, the higher concentrations of MLSS in MBR enhances the color adsorption capacity. Fig. 17, represents the color of feed water and permeate from MBR using CM and fabricated membranes.

PES-PVP blended membrane had the lowest pore size compared to commercial (CM) and PES-PEG membranes thus it exhibited highest percentage of color removal (Fig. 17d).

#### 3.3.3 MBR permeate

**Fig. 18:**
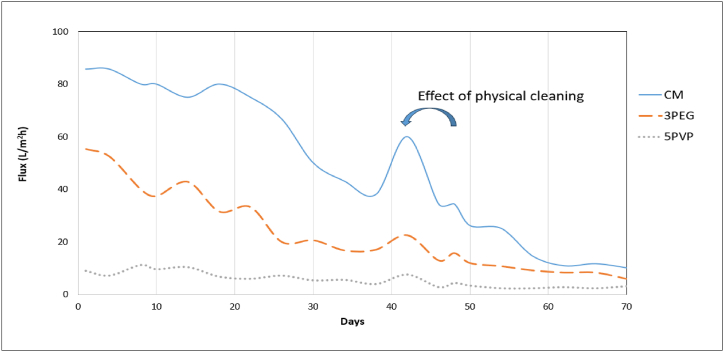
Fluxes of MBR permeate.

Permeate flux from the CM and the PES-3PEG and PES-5PVP membranes were illustrated in Fig. 18. Over the 70 days of operation, the average flux obtained from the CM, PES-3PEG, and PES-3PVP membranes were 39, 18, and 5 L/m^2^h. Initially, all the membranes exhibited higher fluxes which gradually declined over the course of operation due to the fouling of the membranes. On the 39^th^ day, a physical cleaning procedure was executed on the membranes leading to substantial enhancement of the flux and regained. For instance, the initial fluxes of the CM, PES-3PEG, and PES-5PVP membranes were 86, 55, and 9 L/m^2^h respectively which dropped down to 60, 23, and 8 L/m^2^h after 39 days. However, following physical cleaning, these fluxes significantly improved reaching approximately 70 %, 42 %, and 83 % of their respective initial fluxes.

The gradual slow declining of fluxes could be attributed to the incurred irreversible fouling after conducting the physical cleaning on the 39th day. PES-5PVP membrane has the lowest pore size compared to PES-3PES and CM, and as expected, it exhibited the lowest permeate.

#### 3.3.4 Bacteria removal

Bacteria typically exhibit diverse morphologies, adopting spherical (referred to as coccus), rod-shaped (bacillus), and spiral-shaped (spirochete) forms, with diameters ranging from approximately 1 to 2 μm and lengths spanning 5–10 μm [49]. A total of nine (09) bacteria were identified in the MBR sludge with varying counts. Fabricated PES-3PEG and PES-5PVP blended membranes were efficient in removing bacteria (Table 4) due to their smaller pore sizes compared to bacteria. These results were highly comparable to CM membrane, and in certain instances, the fabricated membranes exhibited superior bacterial filtration capacities.

**Table 4:** Percentage of bacteria removal.

Microorganism	Microbiological Removal			
	Raw (CFU/ml)	CM (%)	PES-3PEG (%)	PES-5PVP (%)
Total aerobic bacterial count	170000	91.76	96.00	91.76
Total coliform count	150000	93.67	99.27	91.33
Total faecal coliform count	78000	93.97	99.74	99.36
Total yeast and mold	24000	99.98	95.00	99.17
*E. coli*	<10	89.00	78.00	89.00
*Bacillus* spp.	65000	81.23	84.62	81.54
*Salmonella* spp.	9300	99.91	99.96	99.96
*Pseudomonas* spp.	<10	89.00	78.00	89.00
*Staphylococcus* spp.	300	99	99	99

**Fig. 19:**
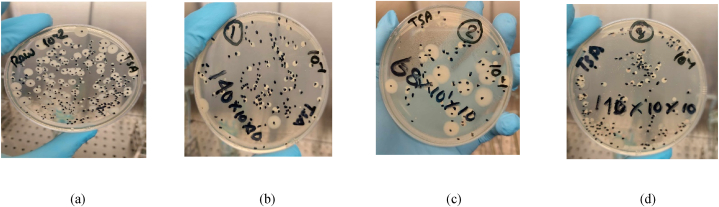
Total aerobic bacteria in (a) raw feed and removal of total aerobic bacteria from (b) CM membrane (c) PES-3PEG membrane, and (d) PES-5PVP membrane.

The efficacy of bacterial removal in the MBR system is enhanced through a combination of biodegradation, biosorption, and membrane retention processes, with size exclusion serving as the main principle for pathogen reduction within the membrane [53,54]. Fig. 19 illustrates the microorganism counts before (a) and after (b-d) treatment.

## 4 Conclusions

PES-PEG and PES-PVP based hydrophilic blended membranes were fabricated employing the NIPS method. PES-PEG blended membrane with 3 wt% PEG showed highest porosity (28.64 %) and pure water flux (1328 L/m^2^h) compared to PES-control membrane which had a 2.73 % porosity and a flux of 226 L/m^2^h. Conversely, PES-PVP blended membrane, at its optimum concentration of PVP (5 wt%), exhibited a porosity of only 15.04 % and a flux of 660 L/m^2^h. On an average, under optimum conditions, PES-3PEG blended membrane exhibited 101 % higher fluxes compared to the PES-5PVP-blended membrane while achieving approximately 82 % of the fluxes exhibited by the PES-based commercial membrane. Regarding hydrophilicity, both blended membranes demonstrated lower contact angles compared to the PES-control membrane.

In MBR applications, PES-3PEG and PES-5PVP blended membranes exhibited 93–94 % BOD, 95–96 % COD removal, and 93–94 % color removal which were significantly comparable with that of PES-based commercial membrane. Despite the disparity in permeate flux, the fabricated membranes (PES-3PEG and PES-5PVP) exhibited significant efficacy in removing microorganisms which was also comparable to that of the commercial membrane.

## Consent to publish

The authors attest that the contents in the manuscript have not been previously published or offered for publication elsewhere.

## Data availability

The datasets generated during and/or analyzed during the current study are available from the corresponding author on reasonable request.

## CRediT authorship contribution statement

**Md. Nur-E Alam:** Writing – review & editing, Investigation, Conceptualization. **Shamim Ahmed Deowan:** Writing – review & editing, Supervision. **Shakil Shahriar Efty:** Investigation. **Fariha Chowdhury:** Investigation. **Ahsanul Haque Milon:** Investigation. **Mohammad Nurnabi:** Writing – review & editing, Supervision, Conceptualization.

## Declaration of competing interest

The authors declare that they have no known competing financial interests or personal relationships that could have appeared to influence the work reported in this paper.
