# Supplementary material for: Fabrication and performance evaluation of polyethersulfone membranes with varying compositions of polyvinylpyrrolidone and polyethylene glycol for textile wastewater treatment using MBR
Source: Heliyon. 2024 Aug 16;10(16):e36215. doi: 10.1016/j.heliyon.2024.e36215 (PMC11380171; doi:10.1016/j.heliyon.2024.e36215)
Supplement: Multimedia component 1 [file mmc1.docx]

**Supplementary information**.

**S1** Solubility parameters of polymers [45,46]

| **Material** | **Solubility parameter**  **δ (MPa^1/2^)** |
| --- | --- |
| Polyethylene glycol (PEG-6K) | 35.3 |
| Polyvinylpyrrolidone (PVP-40K) | 19.4 |
| Polyethersulfone (PES) | 23 |
| DMF | 24.8 |
| Water | 47.8 |
